# Textbook outcomes in oesophageal resections from an evolving thoracic surgical unit

**DOI:** 10.1007/s12055-025-02107-2

**Published:** 2025-12-13

**Authors:** Vishnu Santosh Menon, Rigved Nittala, Amita Sekhar Padhy, Sidaksingh Rajendrasingh Arora, Mounika Basani

**Affiliations:** Department of Surgical Oncology, Homi Bhabha Cancer Hospital & Research Centre, Visakhapatnam, 530053 Andhra Pradesh India

**Keywords:** Textbook outcome, Oesophageal cancer, Surgical quality, Low-volume centre

## Abstract

Textbook outcome (TO) is a composite metric of surgical quality. This study evaluated TO in oesophageal resections at a low-volume thoracic surgical unit in a Tier-2 city in India, assessing its applicability in a resource-constrained setting. This retrospective study included all patients with oesophageal cancer undergoing curative resection at our centre from 1 January 2020 to 31 December 2024. Patients were identified from a prospectively maintained surgical database and electronic medical records. Clinico-radiological, histopathological, and surgical outcomes were evaluated. TO was defined using the 2017 Dutch Upper Gastrointestinal (GI) Cancer Audit, comprising ten parameters, including complete resection, no major morbidity, and adequate lymph node yield. Among 41 patients, TO was achieved in 14 (34.1%). Major perioperative morbidity (Clavien-Dindo Grade ≥ II) occurred in 7 (17.1%) patients, with two (4.8%) perioperative deaths. Complete resection as per surgeon assessment was achieved in all cases (41/41, 100%), while optimal lymph node yield (≥ 15 nodes) was the most challenging parameter (25/41, 61%), followed by no 30-day readmission (34/41, 82.9%). TO was achieved in one-third of patients, comparable to international benchmarks, but optimal lymph node yield remains a challenge. TO is a useful quality assurance tool, though context-specific adaptations may enhance its relevance in India.

## Introduction

Surgery is the cornerstone of treatment for resectable middle and lower third oesophageal cancers, aiming for curative resection and reconstruction [1, 2]. This complex procedure requires expertise across thoracic and abdominal domains [3]. The high-risk nature of oesophagectomy is amplified by neoadjuvant therapies, which improve survival compared to upfront surgery but increases perioperative challenges [4]. Complications range from minor surgical site infections to severe events like anastomotic leakage, pulmonary issues, or cardiovascular events [5, 6]. Globally, centralization of oesophagectomy to high-volume centres has improved outcomes [7, 8]. However, in India, vast geography and disparities in healthcare access, make centralization challenging, necessitating quality surgical care in regional centres [9]. Low-volume centres, particularly in Tier-2 cities, are emerging as critical providers of complex cancer care, yet standardized outcome metrics are lacking [10].

Textbook outcome (TO), introduced by the Dutch Upper GastroIntestinal(GI) Cancer Audit, is a composite measure of ten short-term surgical parameters to assess quality [11]. This study evaluates TO in oesophageal resections at a low-volume thoracic surgical unit in a Tier-2 city in India, aiming to assess surgical quality and the applicability of TO in a resource-constrained setting.

## Methods

### Study design, population, exclusion criteria, and endpoints

This retrospective cohort analysis was conducted at the thoracic surgical unit of a comprehensive cancer centre in India. A prospectively maintained institutional database and the electronic medical record (EMR) from 1 January 2020 to 31 December 2024 were assessed to identify patients undergoing curative oesophageal cancer resections. Inclusion criteria were: (1) Age ≥ 18 years, (2) Stage I-III oesophageal cancer treated with curative intent, (3) Surgery between 1 January 2020 and 31 December 2024. Exclusion criteria included: (1) recurrent disease, (2) prior surgery elsewhere, (3) resection for benign/non-malignant aetiology. The primary objective was to evaluate TO achievement and its applicability in a low-volume, resource-constrained setting. Secondary objectives included identifying factors influencing TO and non-TO status.

### Data retrieval

Clinical data, including demographics, pathological characteristics, surgical details, postoperative outcomes, and neoadjuvant/adjuvant therapies, were collected from the prospective surgical database and EMR. Disease extent was confirmed via preoperative imaging, operative notes, and histopathology reports. Missing data were handled by listwise deletion for variables critical to TO assessment (e.g., lymph node yield, morbidity); otherwise, variables were reported as incomplete.

### Defining textbook outcomes

TO was defined per the 2017 Dutch Upper GI Cancer Audit, comprising ten parameters: (1) complete resection per surgeon assessment, (2) no intraoperative complications, (3) no major perioperative morbidity (Clavien-Dindo Grade ≤ I), (4) intensive care unit (ICU) stay < 24 h, (5) hospital stay ≤ 21 days, (6) no readmission within 30 days, (7) no reintervention within 30 days, (8) no postoperative mortality, (9) R0 resection (tumour-negative margins), and (10) ≥ 15 lymph nodes retrieved [11]. This definition was chosen due to its alignment with our cohort’s median nodal yield (16 nodes) and established validation, though we acknowledge newer definitions [12].

### Surgical technique

Ivor-Lewis oesophagectomy (ILE) and transthoracic oesophagectomy (TTE) included two-field lymphadenectomy (mediastinal and abdominal nodes), targeting paraoesophageal, subcarinal, and perigastric nodes. Trans-hiatal oesophagectomy (THE) involved abdominal and lower paraoesophageal lymph nodes. Video-assisted thoracoscopic surgery (VATS) or laparoscopy was attempted where feasible, with conversion to open procedures as needed.

### Statistical analysis

Data were analyzed using IBM SPSS version 29. Descriptive statistics included medians with interquartile ranges (IQR) for skewed continuous variables (e.g., lymph node yield, hospital stay) and means with standard deviations for normally distributed variables (e.g., age). Categorical variables were reported as numbers and percentages, compared using chi-square or Fisher’s exact tests. Odds ratios (OR) with 95% confidence intervals (CI) were calculated via univariate analysis to identify factors associated with TO. Multivariate analysis was not performed due to the small sample size, which limited statistical power. A p-value ≤ 0.05 was considered significant.

### Ethical considerations

The study was approved by the Institutional Ethics Committee (Project No 12000092) and complied with the 1964 Helsinki Declaration and its amendments. Informed consent was obtained for all surgical and clinical procedures as part of routine practice.

## Results

### Cohort characteristics (Tables 1 and 2)

We identified 41 patients, with a median of 5 cases per year (range 2–16). The median age was 56 years (IQR 48–64). Squamous cell carcinoma comprised 75.6% (31/41) of cases, with 97.5% (40/41) having infra-carinal disease. Neoadjuvant therapy was administered to 37/41 patients (90.2%), including chemoradiotherapy in 30 (73.2%) and chemotherapy in 7 (17.0%). The remaining 4 patients (9.8%) had early-stage disease (T1N0) and underwent upfront surgery. Preoperative *American Society of Anesthesiologists* grade(ASA) ≥ 2 was noted in 7/41 (17.1%) patients, 8/41 (19.5%) were smokers, and 3/41 (7.4%) had increased pulmonary risk. All cases were discussed in multidisciplinary clinics and underwent prehabilitation. ILE was performed in 23/41 (56.1%), TTE in 5/41 (12.2%), and THE in 13/41 (31.7%). VATS was attempted in 19/41 (46.3%), with 7 (36.8%) conversions to open. Laparoscopy was attempted in 20/41 (48.7%), with 1 (5%) conversion. Median blood loss was 420 mL (IQR 300–600) (Tables 1 and 2).

**Table 1:** Baseline cohort characteristics

Parameter	Over all (*N* = 41)	TO (*N* = 14)	Non-TO (*N* = 27)	Odds Ratio (95% CI)	*p* Value
Age (years)					0.19
Mean $\pm$ SD	56.2 $\pm$ 10.2	54.6 $\pm$ 11.8	57.0 $\pm$ 9.4		
Median (IQR)	56 (48–64)	54 (46–62)	57 (49–65)	1.0 (0.9–1.1)	
$\ge$ 65 years	12 (29.3%)	4 (33.3%)	8 (66.7%)	2.0 (0.5–8.1)	0.32
Gender					0.05
Male	20 (48.8%)	9 (45.0%)	11 (55.0%)	1.9 (0.5–6.8)	
Female	21 (51.2%)	5 (23.8%)	16 (76.2%)		
Body mass index					0.12
Mean $\pm$ SD	20.5 $\pm$ 3.6	19.7 $\pm$ 1.4	21.0 $\pm$ 4.3		
Median (IQR)	19.7 (18–22)	19.5 (18–21)	19.8 (18–23)	0.9 (0.7–1.1)	
Haemoglobin (g/dL)					0.73
Mean $\pm$ SD	11.2 $\pm$ 1.6	11.6 $\pm 1.8$	11.0 $\pm$ 1.5		
Median (IQR)	11.1 (10–12)	11.3 (10–13)	11.1 (10–12)	1.0 (0.7–1.4)	
Serum albumin (g/dL)					0.19
Mean $\pm$ SD	3.75 $\pm$ 0.54	3.97 $\pm$ 0.38	3.63 $\pm$ 0.59		
Median (IQR)	3.67 (3.3–4.1)	3.98 (3.6–4.3)	3.60 (3.2–4.0)	1.2(0.5–2.9)	
Smoking status					0.45
Smoker	8 (19.6%)	5 (62.5%)	3 (37.5%)	1.2 (0.3–5.5)	
Non-smoker	33 (80.4%)	9 (27.3%)	24 (72.7%)		
Histopathology					0.20
Squamous cell carcinoma	31 (75.6%)	11 (35.4%)	20 (64.6%)	1.2 (0.3–5.0)	
Adenocarcinoma	10 (24.4%)	3 (30.0%)	7 (70.0%)		

*TO* Textbook Outcome, *IQR* Interquartile Range, *CI* Confidence Interval, *SD* Standard Deviation

**Table 2:** Treatment details

Parameter	Overall (*N* = 41)	TO (*N* = 14)	Non-TO (*N* = 27)	Odds Ratio (95% CI)	*p* Value
T stage (clinico-radiological)					0.77
T1/T2	5 (12.2%)	2 (40.0%)	3 (60.0%)	1.3 (0.2–8.7)	
T3/T4	36 (87.8%)	12 (33.3%)	34 (66.7%)		
N stage (clinic-radiological)					0.11
N0	11 (26.8%)	5 (45.5%)	6 (54.5%)	1.9 (0.5–7.6)	
N1	30 (73.2%)	9 (30.0%)	21 (70.0%)		
Preoperative treatment					0.30
Neoadjuvant (NACT/NACRT)	37 (90.2%)	11 (29.7%)	26 (70.3%)	7.1 (0.7–70.3)	
Upfront surgery	4 (9.8%)	3 (75.0%)	1 (25.0%)		
Surgical procedure					0.41
Ivor-Lewis oesophagectomy	23 (56.1%)	8 (34.8%)	15 (65.2%)		
Transthoracic Oesophagectomy (TTE)	5 (12.2%)	1 (20.0%)	4 (80.0%)		
Trans-hiatal oesophagectomy (THE)	13 (31.7%)	5 (38.5%)	8 (61.5%)		
Abdominal approach					0.46
Open	22 (53.7%)	6 (27.3%)	16 (72.7%)	1.7 (0.5–5.8)	
Laparoscopic	19 (46.3%)	8 (42.1%)	11 (57.9%)		
Thoracic approach					0.33
Open	16 (39.0%)	4 (25.0%)	12 (75.0%)	2.3 (0.6–8.7)	
VATS	12 (29.3%)	6 (50.0%)	6 (50.0%)		
Not applicable (THE)	13 (31.7%)	4 (30.7%)	9 (69.3%)		

*TO* Textbook Outcome, *NACT* Neoadjuvant Chemotherapy, *NACRT* Neoadjuvant Chemoradiotherapy, *VATS* Video-Assisted Thoracoscopic Surgery, *THE* Trans-hiatal oesophagectomy

### Individual parameters (Table 3, Fig. 1)

**Fig. 1:**
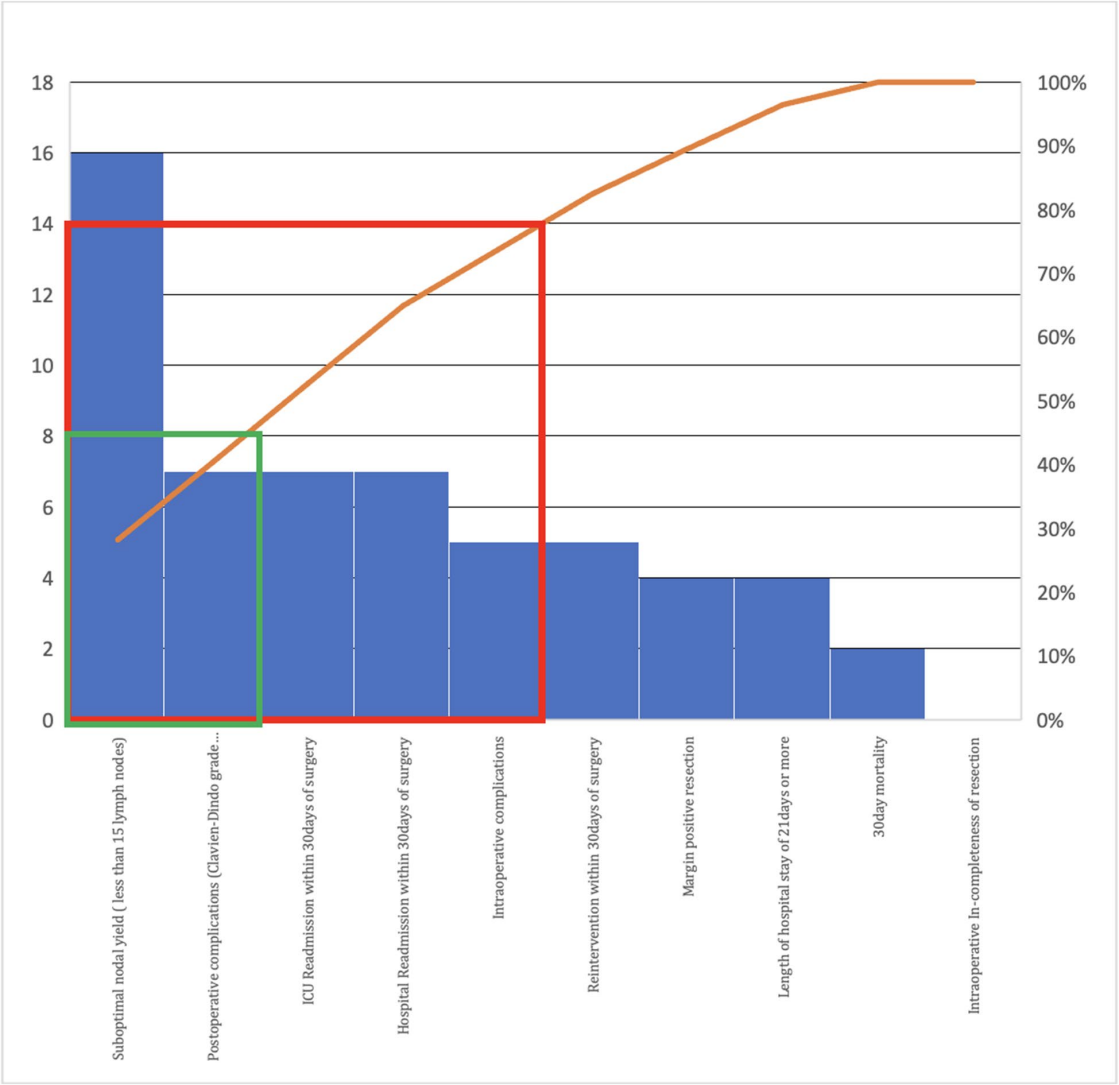
Pareto chart of the non-textbook outcome subset. The left and right side of y axis represents frequency and percentage. The x-axis represents individual parameters. The red line represents the 80% acceptance interval and green line represents the 50% acceptance interval

Complete resection, per surgeon assessment, was reported in 41/41 cases (100%). R0 resection (tumour-negative margins) was achieved in 37/41 (90.2%), with 4 cases having positive circumferential margins (CRM). No proximal or distal margin positivity was noted, and no adjuvant radiotherapy was required for CRM positivity based on multidisciplinary consensus. Intraoperative complications occurred in 5/41 (12.2%) patients: major bleeding (> 1000 mL, adopted arbitrarily) in 2, gastric tube ischaemia in 1, biliary tract injury in 1, and arrhythmia in 1, all managed intraoperatively. Anastomotic dehiscence occurred in 6/41 (14.6%). Prolonged ICU care or readmission was needed in 7/41 (17.1%). Median hospital stay was 12 days (IQR 9–18). Readmission within 30 days occurred in 7/41 (17.1%). Major morbidity (Clavien-Dindo Grade ≥ II) was noted in 7/41 (17.1%), all Grade ≥ III. Two perioperative deaths (4.8%) occurred: one from an acute coronary event (with intraoperative complication) and one post-ILE from anastomotic leakage on day 21. Median lymph node yield was 16 (IQR 12–22), with ≥ 15 nodes achieved in 25/41 (61%). Yield was lower in THE (median 12, IQR 8–16) vs. ILE/TTE (median 18, IQR 14–24). Figure 1 (Pareto chart) illustrates that lymph node yield and readmission were the most frequent barriers to TO (Table 3).

**Table 3:** Treatment outcomes and individual parameters

Parameter	Overall (*N* = 41)	Percentage	Non-TO (*N* = 27)	Non-TO Percentage
Complete resection per surgeon	41	100	27	100
R0 resection (no tumour in margins)	37	90.2	23	85.1
$\ge$ 15 lymph nodes retrieved	25	61.0	11	40.7
No intraoperative complication	36	87.8	22	81.5
No complication $\ge$ clavien-Dindo Grade II	34	82.9	20	74.1
No reintervention $\le$ 30 days	36	87.8	22	81.5
No ICU readmission $\le$ 30 days	34	82.9	20	74.1
Hospital stay < 21 days	37	90.2	23	85.1
No in-hospital/30-day mortality	39	95.1	25	92.6
No readmission $\le$ 30 days	34	82.9	20	74.1

*TO* Textbook Outcome,* ICU* Intensive Care Unit

### TO as a composite outcome

TO was achieved in 14/41 patients (34.1%). Among non-TO patients, 15/27 (55.6%) met 9/10 parameters, indicating near-TO status. The most achievable parameter was complete resection per surgeon (100%), while optimal nodal yield was the least (61%).

### Factors influencing TO

TO patients had lower median Body Mass Index, BMI (19.5 vs. 19.8 kg/m^2^, *p* = 0.12), higher haemoglobin (11.3 vs. 11.1 g/dL, *p* = 0.73), and were younger (median 54 vs. 57 years, *p* = 0.19). Patients ≥ 65 years had higher odds of non-TO (OR 2.0, 95% CI 0.5–8.1, *p* = 0.32). Minimally invasive procedures trended toward TO, but differences were not significant (*p* = 0.46 for abdominal approach, *p* = 0.33 for thoracic).

## Discussion

Oesophageal cancer surgery is high-risk and low-volume, prompting global standardization and centralization [7, 13]. In India, geographical and access barriers necessitate regionalized care [9]. Our centre, with a median of 5 cases annually (range 2–16), is a low-volume unit, though surgical volume increased to 16 cases/year in 2023–2024. This study, the first to evaluate TO in oesophageal resections in South India, achieved TO in 34.1% of patients, comparable to Kalff et al.’s 30.7% in a high-volume setting [14].

Optimal lymph node yield (≥ 15 nodes, achieved in 61%) was the most challenging TO parameter, unlike Kalff et al., where major morbidity was hardest [14]. Nodal yield was influenced by surgical approach: trans-hiatal oesophagectomy (THE, 31.7% of cases) yielded fewer nodes (median 12, IQR 8–16) due to limited mediastinal dissection compared to Ivor-Lewis or transthoracic approaches (median 18, IQR 14–24). Neoadjuvant therapy (90.2% of cases) may reduce nodal yield, as reported elsewhere [15]. Additionally, our learning curve, with only 41 cases over 5 years, likely impacted consistency in lymphadenectomy. Pathologist expertise in node identification also plays a role, suggesting a need for standardized protocols [16]. To address this, we plan to implement surgical and pathological training to enhance nodal yield consistency.

TO’s ten parameters effectively captured surgical quality, though overlaps (e.g., no morbidity vs. no mortality) suggest potential refinement. Our 34.1% TO rate aligns with international benchmarks, validating its use in low-volume settings. The 2017 TO definition includes a 21-day hospital stay threshold. In our Tier-2 city, logistical factors like patient travel, social support might not allow extended stays beyond clinical need, highlighting the need for context-specific adaptations, such as adjusting stay thresholds to reflect local realities [17]. For example, a 14-day threshold, as in newer TO definitions, may better suit enhanced recovery protocols but requires validation in India [12].

Survival analysis was not feasible due to short follow-up, but Kalff et al. linked TO to better survival, suggesting future research priorities [14]. Comparing our findings to other low-resource settings (e.g., Southeast Asia, Africa) could further validate TO’s utility [18]. Strengths include our pioneering low volume Indian centre TO assessment and comprehensive data collection.

## Limitations

Our study is limited by its small sample size (n = 41), retrospective design, and potential selection bias due to the single-centre nature. Missing data in some variables were handled by listwise deletion, which may affect result generalizability. The use of the 2017 TO definition, though aligned with our median nodal yield, may not reflect newer standards. Short follow-up precluded survival analysis, and the learning curve of a low-volume centre may influence outcomes. Future studies should refine TO parameters through multi-institutional collaboration, such as India’s National Cancer Grid, to develop metrics tailored to regional healthcare contexts [19].

## Conclusion

Textbook outcomes were achieved in 34.1% of oesophageal resections, comparable to global standards, with optimal lymph node yield as the primary challenge. TO is a valuable tool for surgical quality assurance in low-volume centres, identifying strengths and areas for improvement. Context-specific adaptations, informed by multi-institutional data, could enhance TO’s relevance in India.

## Data Availability

Data is available on reasonable request from the corresponding author.
